# Smartphone technology, social network maintenance, and social participation among Mandarin-speaking older immigrants in Canada

**DOI:** 10.1093/geront/gnag167

**Published:** 2026-08-03

**Authors:** Prince C Ekoh, Sepali Guruge, Jordana Salma, Zhixi C Zhuang, Bharati Sethi, Christine A Walsh

**Affiliations:** School of Social Work, Department of Social Sciences, University of New Brunswick, Saint John, New Brunswick, Canada; Dibịa Akwụkwọ: Social Solutions Research Group (SSRG), Nsukka, Nigeria; Faculty of Social Work, University of Calgary, Calgary, Alberta, Canada; Daphne Cockwell School of Nursing, Toronto Metropolitan University, Toronto, Ontario, Canada; College of Health Sciences, Faculty of Nursing, University of Alberta, Edmonton, Alberta, Canada; School of Urban & Regional Planning, Education & Capacity Building, Global Migration Institute, Toronto Metropolitan University, Toronto, Ontario, Canada; Department of Political Studies, Trent University, Peterborough, Ontario, Canada; Faculty of Social Work, University of Calgary, Calgary, Alberta, Canada

**Keywords:** Ageing, Digital technology, Social connection, Online activities

## Abstract

**Background and Objectives:**

The rapid global expansion of digital technologies has reshaped everyday life across generations, yet the role of smartphones in older immigrants’ social well-being remains insufficiently understood. Drawing on the sociotechnical perspective that ageing and technology are co-constituted; our study examines how Mandarin-speaking older immigrants in Canada use smartphones to overcome social isolation and sustain social participation.

**Research Design and Methods:**

Data were obtained from 102 qualitative interviews conducted across seven Canadian cities as part of the Inclusive Communities for Older Immigrants (ICOI) project. The data were analyzed using a hybrid deductive–inductive thematic analysis.

**Results:**

3 overarching themes were identified: experiences of social isolation, shaped by linguistic, cultural, and seasonal barriers; smartphones as tools for social connection; and smartphones as facilitators of social participation, enabling both online engagement and the coordination of in-person activities. Study findings demonstrate that smartphones operate not merely as communication devices but as sociotechnical infrastructures that sustain belonging, enable local and transnational connectivity, and support mutual aid among older immigrants. At the same time, they complement rather than replace in-person interaction, with many participants preferring face-to-face social engagement when feasible.

**Discussion and Implications:**

This study contributes to critical gerontology and migration scholarship by illustrating how digital technologies address social isolation among older immigrants and highlights the need for culturally and linguistically tailored digital inclusion initiatives that promote equitable ageing in diverse societies.

The use of digital technology is increasing worldwide; however, scholars are only beginning to understand the implications of smartphone use for their well-being. Early research in technology use for ageing focused on how technology can be used in ageing interventions; however, this study follows the argument of Peine and Neven (2019, 2021) and Emmesjö et al. (2025), who identify digital technology as part of the lived experience of older adults; hence, there is a need for more understanding of the co-constitution of ageing and technology. This study aims to contribute to this growing body of knowledge by examining how Mandarin-speaking older immigrants in Canada use smartphones to maintain social connections.

Historically, older adults have experienced greater digital exclusion and limited smartphone use compared to younger people. However, smartphone use among older adults is increasing globally (Eurostat, 2022a, 2022b; Government of Canada and Statistics Canada, 2021). For instance, the 2021 Statistical Report on Internet Development in China documented a dramatic increase in smartphone use among older Chinese adults, from 5.9% in 2010 to 26.3% in 2020 (Chinese Internet Network Information Centre, 2021). Research in emerging areas demonstrates the diverse ways older adults use smartphones in everyday life in China (Chai et al., 2018). However, little is known about smartphone use among older Chinese adults outside China. Hence, guided by the question: how do Mandarin-speaking older immigrants in Canada use smartphones to address social isolation and disconnection, this study explores how Mandarin-speaking older adults across four Canadian provinces use smartphones to improve social connection and participation.

## Smartphone use and the well-being of older adults

Smartphones, alongside smartwatches and other connected monitoring devices, play a critical role in supporting various aspects of health monitoring among older adults. Among other functions, these devices facilitate the tracking of falls, blood glucose levels, electrocardiograms, and blood pressure, and they promote health-seeking behaviors such as increased physical activity and adherence to medical routines (Marcolino et al., 2018; Odeh et al., 2024; Salma et al., 2025; Wu & Luo, 2019). Furthermore, Liang et al. (2022) demonstrated a positive relationship between smartphone use and self-rated health in older adults. Simple smartphone functions, such as text messaging, have been shown to enhance disease management, improve medication adherence, and increase attendance at regular medical check-ups (Marcolino et al., 2018). Consequently, digital literacy and access to such technologies have been described as “digital determinants of health” (Chidambaram, 2024; Jahnel et al., 2022) or “super determinants of health” (van Kessel et al., 2022).

Emerging evidence also highlights the cognitive benefits of smartphone use. For instance, a systematic review by Wilson et al. (2022) found that smartphone use can enhance cognitive function among older adults without cognitive impairment, as it is considered a cognitively stimulating activity. However, similar effects were not observed among those with cognitive decline. Beyond physical and cognitive health, smartphones have been linked to improved psychosocial well-being. Research consistently links social connectivity, often facilitated through smartphone use, with improved physical health and emotional well-being in later life. Chai et al. (2018) reported that smartphone use increased self-reported happiness among older adults in both urban and rural China. Similarly, during the COVID-19 pandemic, Ikeuchi et al. (2023) found that frequent smartphone users reported feeling younger than their chronological age, due to the association of smartphone use with younger people. Studies by Sagong and Yoon (2022) indicated that older Korean adults who used smartphones reported significantly higher life satisfaction than their non-using counterparts. While the scholars could not identify the rationale for the increase in life satisfaction, they inferred from previous studies that it may be associated with smartphone use, increased engagement in social activities, access to information, interactions with social networks, and self-esteem from acquiring new technology. Research also reveals associations between smartphone ownership and reduced depression (Kim, 2018; Sagong & Yoon, 2018), increased participation in social activities (Briede-Westermeyer et al., 2020; Ekoh et al., 2025, 2026; Sagong & Yoon, 2018, 2022), and overall improvements in lifestyle and care-related outcomes for older adults.

However, while the benefits are well documented, emerging research highlights potential risks. Nahas et al. (2018), Shi et al. (2025), and Xu et al. (2023) identified smartphone addiction as an emerging issue among older adults in China, where excessive use can undermine social connections rather than foster them, leading to social alienation. Similarly, a cross-sectional study by Kim et al. reported smartphone overdependence among some older adults in a survey of 630 older Koreans who use smartphones (Kim et al., 2024). Another Korean study reveals that such patterns of use have been associated with reduced health-related quality of life and an increased risk of cognitive impairments (Jeong et al., 2020). These findings underscore the importance of promoting balanced and moderated smartphone use through targeted education and digital literacy initiatives (Xu et al., 2023).

## Smartphone use and social isolation and loneliness among older adults/immigrants

Social isolation, defined as the objective state of having minimal or infrequent social contact with others, and loneliness, understood as the subjective and distressing experience of feeling socially or emotionally disconnected regardless of the actual level of social interaction, represent significant public health concerns among older adults (Employment and Social Development Canada [ESDC], 2018; Grenier et al., 2022; Statistics Canada, 2023). Approximately 25% of community-dwelling older adults experience social isolation globally (Teo et al., 2023). However, the risk is likely higher among racialized older immigrants who are physically separated from their homes and often face the compounding effects of systemic racism and exclusion (National Seniors Council, 2014, 2017; Statistics Canada, 2023). Such experiences have been linked to a range of adverse health outcomes, including physical health deterioration, mental health challenges, dementia, cognitive impairments, and increased morbidity and mortality (Freedman & Nicolle, 2020; Guruge et al., 2019, 2025).

Digital technology, particularly smartphones, has emerged as a potential tool for social participation, which involves active engagement in society and community life (Hoffmann et al., 2023) as well as maintaining social connections that involve meaningful relationships and interactions with social ties (Ekoh et al., 2025), collectively mitigating social disconnection among older adults. Evidence from the COVID-19 pandemic, during which social distancing and lockdowns heightened social isolation and loneliness, demonstrated that smartphones played a crucial role in enabling older adults to maintain connections with family and friends, thereby reducing feelings of disconnection (Ekoh et al., 2021, 2025; Neves et al., 2018, 2019; Salma et al., 2021). For instance, video calls conducted via smartphones in nursing homes were reported to alleviate loneliness among older residents (Metersky et al., 2025; Tsai et al., 2020). A systematic review by Sen et al. (2022) further emphasized the potential of smartphones in addressing cognitive, visual, and hearing impairments that exacerbate loneliness, thereby facilitating stronger connections between older adults and their social networks. Older immigrants also increasingly utilized smartphones to maintain their social connections during the pandemic, to address loneliness, and to enhance well-being (Ekoh et al., 2023; Rehmani et al., 2023; Sidani et al., 2022; Suntai & Beltran, 2023; Yang et al., 2025).

Among older adults in China, Liu et al. (2022) and Yang and Du (2021) found that smartphone use helped users overcome temporal and geographic barriers, thereby enhancing communication and strengthening social support networks. Conversely, Xie et al. (2021), drawing on data from the China Longitudinal Ageing Social Survey, reported that increased smartphone use was associated with reduced in-person social interactions and contributed to heightened depression among older adults. Similarly, Cui et al. (2024) and Shi et al. (2025) found that excessive reliance on WeChat© (a super app with communication, shopping, and other features), often driven by Fear of Missing Out and sensation-seeking behaviors, was associated with increased loneliness and social isolation in this population. Despite these insights, there remains a notable gap in researchers’ understanding of how smartphone use influences social isolation among diasporic Chinese older adults across Canada, a population growing at a dramatic rate. According to the 2021 census data, Mandarin is the fourth most spoken language in Canada, with 987,000 speakers (Government of Canada, 2021). Furthermore, Chinese older adults represent about 28% of all racialized older adults in Canada (Ferreira, 2023). A likely notable difference between migrant and non-migrant Chinese older adults is the use of smartphones and other information and communication technologies by migrants to maintain transnational social connectedness and exchange information, with their social ties across international borders (Baldassar & Wilding, 2020; Nguyen et al., 2022). While this is a growing body of literature, there is little evidence on how transnational interactions are specifically shaping the digital activities of older Chinese migrant adults (Ekoh et al., 2025). This study aims to address this gap by examining how Mandarin-speaking older immigrants in seven Canadian cities utilize smartphones to foster or hinder social connectedness, thereby contributing to the broader discourse on ageing, migration, and digital inclusion.

## Method

### Research context

Grounded in an interpretivist qualitative framework, this study prioritizes the exploration of subjective meanings, lived experiences, and the social contexts that shape human understanding. Adopting this interpretive lens enabled an in-depth exploration of how older immigrants understand, negotiate, and make meaning of their everyday experiences, relationships, and care practices within their sociocultural environments. This study is part of a larger, multi-phase project entitled Inclusive Communities for Older Immigrants (ICOI). The overarching objective of this project is to examine the multifaceted factors contributing to social disconnection and to develop strategies that promote social inclusion among older immigrants in Canada. Data for the ICOI project were collected across seven cities situated in four Canadian provinces: Quebec (Montreal), Ontario (Toronto and London), Alberta (Calgary and Edmonton), and British Columbia (Vancouver and Victoria). Focusing on language groups, the broader research examines three of the largest immigrant populations in Canada, Mandarin-, Punjabi-, and Arabic-speaking older adults, and seeks to identify culturally responsive interventions to address the risk of social isolation among these groups.

### Recruitment

Participants were recruited primarily through established community partnerships with immigrant-serving agencies and cultural community centers. However, in general, each city used a combination of active and passive strategies, including distributing study flyers written in Mandarin and English at immigrant-serving agencies, relying on word-of-mouth referrals, and engaging potential participants directly through community events and agency contacts. This recruitment strategy facilitated access to potential participants while ensuring cultural sensitivity and trust-building. Inclusion criteria were as follows: participants had to be 60 years of age or older, foreign-born, residing in one of seven cities at the time of the study, and able to provide informed consent. A total of 102 participants were recruited (Calgary = 10, Edmonton = 11, London = 35, Montreal = 10, Toronto = 15, Vancouver = 10, and Victoria = 11). This sample size ensured data completeness.

### Data collection

Ethical approval for this study was obtained from the relevant institutional research ethics boards in each city prior to data collection. Data were collected with semi-structured interviews designed and approved by the national team. Each interview lasted between 35 and 60 min and, with participants’ consent, was audio-recorded. With participants’ consent, interviews were audio-recorded. Recordings were subsequently translated (where necessary) and transcribed. To ensure linguistic accessibility and cultural appropriateness, participants were given the option to conduct their interviews in English or Mandarin. Bilingual and bicultural research assistants (RAs) fluent in Mandarin and English, trained in qualitative interviewing techniques, facilitated the interviews. The RAs were trained in ethical and safe recruitment and data collection before going to the field. The research leaders and RAs met every two weeks to debrief on data collection and share ideas to improve the process. All authors have backgrounds in gerontology and migrant health and training in qualitative methods. Additionally, some of the authors were cultural and linguistic insiders, and collectively they mentored the RAs on this project, providing opportunities for reflexivity and addressing research issues as they arose. Data collection was conducted either face-to-face at community centers familiar to the participants, thereby creating a comfortable and accessible environment for participation or virtually, depending on participants’ preferences. All participants provided informed consent and were assured of the confidentiality and anonymity of their responses.

### Data analysis

The research assistants who are proficient in Mandarin and English translated and transcribed the data. The transcribed data were further reviewed by team members who are also fluent in Mandarin and English to ensure completeness and validity of the transcripts. Data analysis employed a hybrid deductive–inductive thematic strategy, facilitated by NVivo 14© to support systematic coding and data management. The national research team first developed a preliminary codebook, informed by preliminary findings and relevant scholarship on ageing, migration, and digital inclusion. This codebook served as a deductive scaffold and was disseminated to all participating research sites. Building on this framework, each city team conducted inductive, line-by-line coding to allow unanticipated ideas, meanings, and patterns to emerge organically from participants’ narratives.

Coding proceeded by constructing hierarchical coding trees, in which broad conceptual categories served as grandparent nodes, with parent and child nodes capturing progressively refined subdimensions of participants’ experiences. Codes were iteratively reviewed, compared across sites, and reorganized to enhance conceptual clarity and analytic depth. Following Braun and Clarke’s (2023) principles of reflexive thematic analysis, coded segments were synthesized into candidate themes. We had multiple rounds of reading and discussing the transcripts before developing initial codes. We then sorted our codes into preliminary themes and subthemes based on ideas and patterns. These preliminary themes underwent multiple rounds of team-based interrogation, during which themes that were too diffuse, conceptually overlapping, or weakly evidenced were collapsed or redefined to ensure coherence, internal consistency, and strong empirical grounding. The final themes were refined to ensure the scope of each theme is maintained during the writing.

While the research team is diverse, with scholars of South Asian, East Asian, Middle Eastern, African, and European descent, Mandarin-speaking team members took a leading role in interpreting and analyzing the data. To strengthen the credibility and cultural validity of the analysis, member checking was conducted with the broader research team, led by bilingual and bicultural team members who understood the nuances of the narratives. The Mandarin-speaking team members reviewed the final thematic interpretations for linguistic accuracy and cultural resonance. This collaborative process ensured that the final themes faithfully represented participants’ intended meanings and the sociocultural contexts that shaped their accounts. In reporting the findings, participant numbers, city, age, gender (F = female, M = male), and immigration status (C = citizen, P = permanent resident, I = temporary immigrant) were used to label participants. For example, the label “Participant 2, Victoria, 70, F, C” represents a 70-year-old (F) female participant from Victoria who is a (C) citizen and was the second participant interviewed.

## Results

Results show three main themes and six subthemes. As shown in Table 1, the first theme sets the stage by offering a snapshot of how Mandarin-speaking older immigrants in Canada experience social isolation. The second theme demonstrates how the participants use smartphones for social connection across four domains: local communication, transnational connections, online information that facilitates connection, and social support from virtual connections. The final theme focuses on how smartphones facilitate both online and offline social participation among participants.

**Table 1:** Themes and sub-themes.

Themes	Sub-themes
Experience of social isolation	
Smartphones as tools for social connection and belonging	Smartphones as a lifeline for local communication
	Transnational connections
	Online information and social interaction
	Social connection leading to support
Smartphones as a tool for social participation	Participation in online social activities
	Facilitation of in-person social activities

### Participants’ demographics

Table 2 shows participants’ demographic information. The sample consisted of *102 Mandarin-speaking older immigrants*, aged *60 to 92 years*, with a mean age of 71.44 (*SD* = 7.24). Years of residence *in Canada ranged from 1 to 57 years, across seven cities*. The gender distribution was slightly skewed toward females (*58 women, 44 men*). Most participants were *married* (*n* = 63), followed by *widowed* (*n* = 27), *divorced* (*n* = 7), and *separated* (*n* = 5). Regarding employment status, some older immigrants report having retired from formal employment but still engage in menial jobs, thereby distinguishing themselves from fully retired participants.

**Table 2:** Demographic characteristics of participants.

Characteristic	Category	*N*
Gender	Female	58
	Male	44
Age range	60–92 years	—
Years in Canada	1–57 years	—
Provinces	Alberta	21
	British Columbia	21
	Ontario	50
	Quebec	10
Marital status	Married	63
	Widowed	27
	Divorced	7
	Separated	5
Immigration status	Immigrant	8
	Permanent resident	58
	Citizen	36
Employment status	Fully retired	80
	Partially retired	5
	Employed	7
Education level	Bachelor’s degree	25
	Diploma	20
	Completed secondary education	18
	Some secondary education	14
	Master’s degree	8
	Primary education	2
	Other	15

### Experience of social isolation

Social isolation in later life, and its adverse effects on physical, emotional, and cognitive well-being, are well documented in the gerontological literature (Guruge et al., 2025). These risks are often amplified for older immigrants, who must navigate unfamiliar sociocultural environments where linguistic barriers, limited social networks, and differing interaction norms can hinder their ability to form meaningful relationships (Statistics Canada, 2023). While many participants did not disclose social isolation in clear language, consistent with broader evidence, some participants in this study described experiences of social isolation, particularly those living alone and those residing in neighborhoods with minimal linguistic or cultural familiarity. Although participants frequently perceived their neighbors as kind and well-intentioned, meaningful interaction was described as constrained by communication difficulties and concerns about potential cultural misunderstandings. As two participants noted,

With Canadian neighbors, though I don’t interact much due to the language barrier, our interactions are always pleasant. We exchange smiles and greetings. I’ve found that even with limited English, kindness and politeness go a long way in making connections. (Participant 7, London, 79, F, P)

Participants also highlighted the seasonal and cyclical nature of their social relationships. Opportunities for informal outdoor interaction increased in the summer months but declined in the winter, as colder weather reduced mobility and many older Chinese immigrants temporarily returned to China. These patterns contributed to fluctuations in isolation levels throughout the year and were more prevalent among older Mandarin-speaking immigrants in areas with severe and prolonged winters, such as Calgary, Edmonton, and London. A participant from London elucidated:

I usually go out to the public area in our community. It’s a pleasant space where I can breathe in some fresh air and socialize with friends. However, in the later part of the year, as many of my friends return to China for the winter, the neighborhood becomes quieter. (Participant 1, London, 74, F, P)

Even when they remain in Canada during the winter, they limit their movement due to snow and cold. This limits their opportunities to go out and interact with others. A participant from Calgary narrated:

To be honest, the elderly will be worried in winter, and they don’t go out anymore because the snow here is relatively heavy. My old man [husband] broke his arm when he went out in the ice and snow, so he sprinkled water on it. I don’t go out much either. I broke my hand twice on snowy days, you know? I tried it twice, fell twice. Because it was snowing, it was snowing. But if you don’t go out, you’ll be very bored at home all the time. (Participant 1, Calgary, 73, F, P)

In addition, several participants reported that the long-term effects of the COVID-19 pandemic further disrupted established socialization routines, significantly reducing opportunities for in-person gatherings and reinforcing reliance on remote communication.

Yes, we keep in touch. If everyone has something happy to do, we will get together. Before COVID-19, it was normal for us to have gatherings. After the new coronavirus, there were fewer gatherings. This year, it has gradually returned to normal. In the past two years, there were not many gatherings. We usually communicate by mobile phone. At that time, we usually called to say hello and chat. (Participant 2, Edmonton, 77, F, P)

Social isolation was a significant concern for participants due to linguistic barriers, limited social networks, and unfamiliar cultural norms that hindered connectivity with neighbors and the broader non-Chinese community. Social engagement was also seasonal and disrupted by winter conditions and the lingering effects of the COVID-19 pandemic. However, as will be demonstrated below, participants have largely been successful in using smartphones to address their experiences of social isolation.

### Smartphones as tools for social connection and belonging

Against this backdrop of limited in-person interaction, smartphones emerged as a critical resource for maintaining social ties, mitigating isolation, and fostering a sense of belonging among Mandarin-speaking older immigrants. This section presents findings on the role of smartphones in addressing social isolation among participants.

#### Smartphones as a lifeline for local communication

More than half of the participants (55) reported using smartphones to maintain connections with their family and friends in Canada. Participants consistently described smartphones as their primary means of maintaining relationships with family and friends in Canada, most commonly through WeChat and telephone calls. “Mainly through WeChat and phone calls. Friends who live nearby will meet. These are the ways to contact” (participant 5, Edmonton, 65, F, P).

I use phone, WeChat, and online messaging. My friend and I sometimes visit each other. I will visit friends whom I met here, and we will have a meal and drink together. I have played mahjong [a traditional Chinese game] with friends for a little while, but we don’t do that anymore. (participant 32, London, 60, M, C)

These platforms enabled regular communication, coordination of visits, and the cultivation of ongoing companionship. However, while digital communication provided an essential social lifeline, participants continued to express a clear preference for face-to-face interactions whenever feasible, underscoring the complementary, not substitutive, role of digital technologies in fulfilling their social needs.

I mainly stay connected with my family, who I live with, so we’re in constant contact at home. For friends and extended family, I rely on phone calls and, occasionally, social media platforms, though I prefer face-to-face interactions. My grandchildren have taught me how to use some simple apps on the phone to video call my friends and relatives who are far away. (Participant 1, Victoria, 60, F, C)

Smartphones also facilitated the planning and coordination of physical meetings, allowing participants to schedule coffee visits, organize walks, or arrange informal gatherings. This was critical, as the weather can lead to event cancellations during the winter season. In this way, digital tools functioned both as mechanisms of virtual communication and as enablers of offline social engagement.

When I meet neighbors, I chat with them. With relatives, I occasionally make phone calls. But if I want to meet friends for coffee, I send a message, set a time, and then we meet up for coffee, walking there. (Participant 10, Vancouver, 72, M, C)

However, while digital communication provided an essential social lifeline, participants continued to express a clear preference for face-to-face interactions whenever feasible, underscoring the complementary, not substitutive, role of digital technologies in fulfilling their social needs.

#### Transnational connections

A defining feature of participants’ smartphone use was its role in sustaining transnational social networks. These transnational ties were essential for maintaining continuity in identity, belonging, and emotional support, particularly given the geographical distance from long-standing social networks. Many older immigrants relied on WeChat, Line©, and other messaging apps to maintain regular contact with relatives and friends in China “Through WeChat and phone calls, my wife and I contact relatives and friends in China once every two days” (Participant 4, Edmonton, 71, M, C). Smartphone messaging apps like WeChat and Line were the most widely used, as they are cheaper for messaging and calling internationally than international phone calls. Two participants from Ontario narrated:

They [people on her WeChat group] are my classmates in college, and also those studying medicine. They are all retired, okay? Then on WeChat, I talked about the military academy, especially those where everyone communicated with each other. (Participant 8, Calgary, 79, F, P)

Many of my relatives are in Shenyang, China. We use smartphones and keep in touch via WeChat or through other methods. For example, they also have some YouTube channels or predominantly Bilibili channels (a famous Chinese video-watching platform), so people can share things they are interested in. I also attend some online meetings, but they’re informal, and people just share information. (Participant 2, Toronto, 62, M, C)

Another participant, who uses the WeChat Video visual calling option and also records and shares pictures and videos with her social networks, added:

For my family in China, I will use WeChat and use WeChat FaceTime to talk to them. What I also do is record and share videos. For example, I talk to my university classmates once in a while. We will ask each other to record and share some videos on WeChat, and we are shocked by how old we look, since we are still pretty young in each other’s memories. If someone doesn’t want to use FaceTime, we can call each other. All these things can be done on WeChat, which is very convenient. We can even share pictures. Because of the time zone difference, we generally prefer sharing videos, but we can also do FaceTime if we schedule a time beforehand. But I don’t do this with all my friends; only close friends or those who wish to FaceTime with me. (Participant 24, London, 72, M, P)

Participants not only used smartphones to maintain social connections on social networks in China, but also connected with family members in other countries “The people I keep in touch with the most frequently and directly are my husband and my daughter, although my daughter is not here, but she is in the United States, and we use WeChat” (Participant 6, Edmonsto, 66, F, P). Another participant who grew up in Malaysia and migrated to Canada in his 20s added:

We have been away for a long time. I was in my early 20s when I came, so I don’t have too many connections with Malaysia. But I do talk to my family [in Malaysia]. We do email, iMessage, or phone calls. (Participant 2, Victoria, 70, F, C)

The narratives show that while migration creates distance between Mandarin-speaking older immigrants and their social ties, smartphones have provided an avenue for participants to maintain social connections.

#### Online information and social interaction

Smartphones also played an essential role in facilitating access to information about Canada and global events, which in turn supported social interaction. Participants frequently shared news, discussed current affairs with family members, and exchanged perspectives with friends via WeChat or at family meals. A participant discussed how online news facilitates interactions with family members during dinner:

We often have discussions about current affairs at the dinner table with my family. I wouldn’t say we discuss these issues very frequently, but whenever something significant comes up in the news, we tend to share our views on it. Most of the time, these discussions are informal and just between us family members. (Participant 9, London, 74, F, I)

Other participants described discussing news both in-person and online with family and friends: “Yes, when I make a post about Canada in my WeChat moments, my friends will ask me about Canada, and we will talk.”

I discuss social, economic, and political issues with my family members at the dinner table or with my friends or classmates on WeChat, as they are closely related to our living environment. Some political and economic issues are important to us, such as economic development in China, which is close to our hearts, as we know it will ensure a better quality of life and treatment. (Participant 7, Montreal, 75, M, P)

Consuming online news not only helped participants remain informed but also generated conversational material that strengthened both online and offline communications. This underscores the multifaceted role of smartphones in shaping social engagement, functioning as tools for information access, cultural orientation, and relational maintenance.

#### Social connection leading to support

These smartphone-supported social networks served as critical sources of practical and emotional support. Participants described receiving immediate assistance from adult children when needs arose and noted the reliability of these familial support systems in addressing daily challenges such as grocery shopping or managing household tasks. One participant shared how they receive immediate support from their children via the phone:

Sometimes, when there’s a strong need, I’ll give my son a call. He comes over two or three times a week. If there’s no urgent matter, I patiently wait. When the need is strong, I just call my son. He will bring things over. His home is quite close to [a grocery store], and he often helps me buy groceries. He can get everything, from oil and salt to sauce and vinegar. He is a good son. As long as I have a need, he takes care of it immediately. There’s never a refusal. He’s very proactive, and my daughter-in-law is also nice. (Participant 9, Vancouver, 69, F, P)

Importantly, this support was reciprocal. Participants frequently engaged in mutual aid within their digital communities, helping coordinate childcare, sharing information, and responding to others’ needs within WeChat groups. These reciprocal exchanges illustrate how digital communication technologies can strengthen community-oriented forms of support and reinforce collective resilience among older immigrants and their families. A participant described below how they shared needs and solutions to needs in their WeChat group:

I mean, I don’t know them very well, but everybody’s fine. We always help each other out. For example, in my daughter’s WeChat Moments, friends sometimes ask for help and let us know who can’t pick up their child today, and everyone will help pick up the child. Everybody’s really nice. (Participant 4, Victoria, 65, F, I)

These narratives demonstrate that smartphones are a critical tool for mitigating social isolation by enabling communication with family, friends, and broader social networks both locally and transnationally. Through platforms such as WeChat and telephone calls, participants maintained regular contact, coordinated in-person gatherings, and sustained emotional ties with relatives and friends in Canada and beyond. Importantly, smartphone-supported social networks facilitated reciprocal support, both receiving assistance from family and others and offering help within digital community groups, illustrating how mobile technologies enhance not only social connection but also collective well-being and resilience among older immigrants.

### Smartphones as a tool for social participation

Smartphones emerged as a central modality for enabling social participation among Mandarin-speaking older immigrants in this study. Approximately one-quarter of the participants emphasized the critical role of smartphones in facilitating both online and in-person engagement. In this section, an in-depth analysis of how smartphone use enhances social participation and, in turn, fosters social connectedness, inclusion, and overall well-being within this population is presented.

#### Participation in online social activities

Participants frequently reported organizing and engaging in online social activities such as dancing, gaming, and practicing Tai Chi primarily through Zoom and WeChat. These digital platforms served not only as channels for social interaction but also as sites of informal learning that supported settlement and integration. For many recent immigrants, online programs served as accessible avenues for building technological literacy and acquiring essential knowledge to navigate life in Canada. As one participant explained: “I take online classes. This saves some transportation time” (Participant 6, Edmonton, 66, F, P). Another participant from Toronto similarly highlighted the transformative role of online classes in enhancing their ability to use smartphone features to support daily functioning and settlement: “I am very glad to participate in the Zoom classes organized by the community.” I learned a lot from these classes, including information about Canada, senior benefits, and how to use smartphone apps like Google Translate and Google Maps. It helped me a lot in my daily life. (Participant 15, Toronto, 72, M, P).

The analysis further indicates that online participation was particularly beneficial for the “oldest-old” (aged 80 and above), individuals living with chronic illnesses, frailty, or mobility impairments and those living in the coldest parts of the country with long winters. For these participants, digital engagement reduced the physical burden of attending community programs while enabling consistent participation in social and recreational activities. One participant emphasized:

There are lots of programs every day, such as exercises, games, and Tai Chi. Most programs are through Zoom because we are over 85 years old and not good at going out for programs. We also get information from the Internet and WeChat. (Participant 9, Toronto, 85, M, P)

Despite these advantages, several participants expressed a continued preference for in-person engagement whenever possible. Online participation was often understood as a pragmatic alternative rather than an ideal form of social interaction. The following narrative illustrates this tension, highlighting how digital participation is shaped by practical constraints, such as distance, and physical limitations, including eye strain:

It depends on the distance. If they are far away, I will meet them online; if they are close by, we will arrange it in advance and meet in person. I only joined the [community] community center, and the staff there provided me with information about recreational activities. Yet I only participate in a few because I prefer attending activities in person rather than virtually. I have difficulties joining Zoom meetings, and my eyes cannot tolerate staring at a screen for long. My eyes will fill with tears uncontrollably. (Participant 7, Toronto, 68, F, I)

#### Facilitation of in-person social activities

In addition to enabling virtual engagement, smartphones played a central role in facilitating in-person social activities. Participants commonly used phone calls and WeChat group messaging to coordinate social gatherings, including arranging meeting times, locations, and transportation. These platforms supported mutual aid by enabling group members to identify individuals who might require transportation or additional assistance to participate, especially during the winter. As one participant described:

I usually use WeChat. The group I participate in will share activity information in the WeChat group—time, location, whether pick-up and drop-off are needed, and other details. The Chinese are united in this respect and take good care of the elderly in the group. If elderly people are unable to attend the event on their own, they can ask someone to pick them up, and the event organizer will send a car to pick them up. (Participant 22, London, M, 60, P)

Participants also used WeChat to schedule regular group activities, such as Tai Chi, dance sessions, or outings, that facilitated both social interaction and physical activity. These practices strengthened social networks while promoting health and well-being: “We used to call each other, but now we can make phone calls on WeChat as well. We schedule hangouts. We play Tai Chi at 9:30 when the weather is good and meet in a nearby park” (Participant 28, London, 68, F, C). Another participant elaborated on how WeChat supports coordination of a wide range of leisure and community activities:

I mainly communicate with my old Chinese friends through WeChat and phone calls. We gather to play cards, dance, sing, and join self-organized club activities. For activities like picking vegetables or foraging for wild greens, we often go together. I also attend a women’s center and a seniors’ club where we have weekly activities, though these have been less frequent due to the pandemic. (Participant 1, Montreal, F, 66, P).

The narratives illustrate that smartphones are essential tools for enhancing social participation and well-being among Mandarin-speaking older immigrants by facilitating both online and in-person engagement. Through platforms like Zoom and WeChat, participants joined online activities such as Tai Chi, dancing, games, and community-run classes that supported social connection and informal learning. Despite these advantages, many still preferred in-person interactions, using smartphones to coordinate gatherings, share information, and arrange transportation. These communication practices strengthened mutual aid, supported regular social and recreational activities, and fostered a sense of inclusion and connectedness within their communities.

## Discussion

This study examined how Mandarin-speaking older immigrants in four provinces use smartphones to navigate social isolation, sustain transnational relationships, and participate in social life. Consistent with research documenting heightened risks of social isolation among older immigrants (Gerlach et al., 2024; Guruge et al., 2025), the participants described substantial barriers to forming meaningful relationships with their neighbors in Canada, including language barriers, unfamiliar cultural norms, seasonal constraints, and the lingering effects of the COVID-19 pandemic. These factors collectively reinforced the need for alternative avenues of social engagement. Smartphones, therefore, functioned as critical mediating technologies that helped participants compensate for reduced local networks and enabled ongoing relational continuity within and beyond Canada. While a previous study in Canada suggests potential use of smartphones to develop new connections (Ekoh et al., 2025), most participants in this study used smartphones to maintain social connections with family and friends.

Recent research demonstrates that virtual platforms are one of the three forms of co-presence [physical, virtual, and mediated co-presence] that can address social disconnect amongst older adults (Rottenberg et al., 2023; Sethi et al., 2022; Świdrak et al., 2021), with many older adults preferring virtual co-presence in the absence of physical co-presence. Hence, participants did not perceive smartphones as replacements for in-person relationships but rather as complementary tools that allowed them to maintain regular contact and coordinate face-to-face meetings. This finding aligns with studies suggesting that digital communication can enhance rather than diminish in-person relationships among older adults (Ekoh et al., 2025; Sagong & Yoon, 2022). The reported preference for face-to-face interaction underscores that digital technologies may be most effective when embedded within a broader social environment rather than viewed as stand-alone solutions.

A key contribution of this study lies in its detailed account of how Mandarin-speaking older immigrants in Canada use smartphones to sustain transnational social ties. Maintaining relationships with family and friends in China and, in some cases, other countries helped participants preserve a sense of belonging, continuity, and cultural orientation, elements known to be protective against loneliness and social exclusion in later life (Liu et al., 2022). Through platforms such as WeChat, participants held regular video calls, exchanged media, shared everyday experiences, and engaged in group interactions. These practices resonate with scholarship on “ambient co-presence,” wherein digital interactions create a sense of ongoing mutual awareness that supports emotional closeness across distance (Madianou, 2016).

Participants’ accounts illustrate how smartphones foster both virtual and in-person participation in social, recreational, and educational activities. Online classes, particularly those related to settlement, digital literacy, and health, enabled older immigrants to acquire information that supported daily functioning. Notably, the benefits of online participation were particularly salient for the oldest-old and individuals living with chronic illness. For these groups, smartphones enabled meaningful engagement without the physical burdens of travel. Yet, as in other studies (Kim et al., 2024; Marcolino et al., 2018), participants also expressed a preference for in-person programs when feasible, citing digital fatigue, eye strain, or an emotional preference for physical proximity. These findings highlight that digital participation cannot fully substitute embodied forms of sociality; instead, hybrid or blended models may best support the diverse needs of older immigrants.

Smartphones also enabled robust in-person activities through group messaging, facilitating coordination, transportation arrangements, and mutual aid. The presence of culturally specific WeChat groups underscores the importance of community-organized digital spaces in fostering collective participation and bolstering access to culturally relevant activities. The reciprocity embedded in participants’ smartphone-facilitated networks points to a broader communal orientation characteristic of many Chinese cultural practices. Participants described using smartphones not only to seek support but also to offer assistance, share information, and participate in community problem-solving. These findings show that digital technologies can amplify collectivist forms of caregiving and mutual aid among immigrant groups (Nguyen et al., 2022; Wilding et al., 2022). Again, this reciprocal support challenges narratives that depict older adults as passive users of technology or as recipients of care alone. Instead, Mandarin-speaking older immigrants emerged as active contributors within their digital communities, offering social, emotional, and practical resources to others.

The findings of this study improve our understanding of the role healthy smartphone use plays in practical efforts to address social isolation and loneliness amongst older immigrants. Scholars such as Neves et al. (2019), Sen et al. (2022), and Tsai et al. (2020) have consistently demonstrated that smartphones play a significant role in helping older adults stay connected. However, our study shows that this connection is not passive or pedestrian, but a critical tool for information sharing, social participation and engagement. Smartphones provide opportunities to organize and participate in online activities; some of these activities, like tai chi, are guided by social network connections and lead to physical activity. Furthermore, smartphones did not replace the need for physical engagement but facilitated engagement, helping older immigrants organize and stay informed about physical activities.

### Implications for practice, policy, and research

The findings hold significant implications for community program design, immigrant settlement supports, and digital inclusion policies. First, culturally and linguistically tailored digital literacy initiatives are essential, particularly given participants’ reliance on WeChat, a Mandarin-language platform. Second, hybrid models that combine online instruction with in-person activities may best address the preferences and accessibility needs of older immigrants. Third, service providers should recognize the importance of transnational ties for emotional well-being and develop programs that support such global forms of belonging. Future research could integrate digital ethnography, usage tracking, or longitudinal designs to capture dynamic changes in smartphone practices over time. Future research should further examine smartphone use among older immigrants with limited educational attainment and those from other cultural/linguistic backgrounds. Further comparative work across immigrant groups would also help illuminate how cultural norms shape technology use in later life. Finally, these results highlight the need for digital equity interventions that address affordability, linguistic accessibility, and age-friendly design, particularly for recent immigrants who may lack prior digital exposure.

Given participants’ extensive reliance on smartphones, particularly WeChat, policymakers and service providers should recognize digital technologies as critical tools for addressing social isolation, enhancing access to information, and facilitating community participation. Culturally and linguistically responsive digital inclusion initiatives, including Mandarin-language digital literacy training and technology support programs, can help older immigrants maximize the benefits of smartphone use while reducing barriers related to language and technological confidence. Community organizations, settlement agencies, and health and social service providers should integrate smartphone-based communication strategies into their outreach and programming. For example, WeChat can be used to disseminate information about community activities and social support resources in culturally accessible formats. Hybrid service models that combine virtual and in-person engagement may be particularly beneficial for older adults facing mobility limitations, chronic health conditions, transportation challenges, or seasonal barriers associated with Canadian winters. By leveraging technologies that older Mandarin-speaking immigrants already use and trust, policymakers and practitioners can strengthen social connectedness, reduce loneliness, improve access to services, and promote healthy ageing within immigrant communities.

### Limitations and strengths

A significant strength of this study is its large, multisite sample across multiple cities and provinces, which provides a rich and diverse picture of smartphone use among Mandarin-speaking older immigrants. The inclusion of bilingual and bicultural researchers enhanced cultural validity and interpretive depth. Nevertheless, the study relies on self-reported data, which may be influenced by recall or social desirability biases. Future research could integrate digital ethnography, usage tracking, or longitudinal designs to capture dynamic changes in smartphone practices over time. Future studies can also compare urban versus non-urban, immigrant versus native, and gender differences in smartphone use. Again, it is essential to note that the study participants have a significantly higher level of education, which affects older adults’ smartphone use (Anderson & Perrin, 2017). Again, our study failed to fully explore the risks associated with smartphone use, especially among Mandarin-speaking older immigrants who use smartphones to mitigate against loneliness. Future research on this can investigate the inherent risks and ways to mitigate them to ensure the safe use of smartphones by Mandarin-speaking older immigrants.

## Conclusions

Overall, this study demonstrates that smartphones play a central and multifaceted role in the social lives of Mandarin-speaking older immigrants in Canada. Far from being peripheral or optional tools, smartphones have become integral mediators of communication, transnational belonging, social participation, and mutual support. A key contribution of this study is its examination of the role of smartphones in bridging the migration gap, enabling older immigrants to maintain co-presence with their social networks post-migration. These findings reinforce the need to conceptualize ageing and technology as intertwined processes and underscore the importance of culturally responsive digital inclusion strategies that honor the lived realities of immigrant older adults. Hence, policies and practices must leverage smartphones and similar technologies to help older immigrants build and maintain social relationships that mitigate loneliness and social isolation.

## Funding

This manuscript was submitted as part of the work for the Inclusive Communities for Older Immigrants (ICOI) project. The project was supported by a Social Science and Humanities Research Council of Canada (SSHRC) Partnership Grant [#895-2020-1022]. Please visit https://www.icoi.ca to learn more about the project.

## Conflicts of interest

None declared.

## Data Availability

The data supporting this study cannot be shared openly due to privacy and ethical restrictions. De-identified data are available from the principal investigator upon reasonable request.
